# Economic and technical analysis of an HRES (Hybrid Renewable Energy System) comprising wind, PV, and fuel cells using an improved subtraction-average-based optimizer

**DOI:** 10.1016/j.heliyon.2024.e32712

**Published:** 2024-06-08

**Authors:** Yanjun Wang, Xiping He, Qiang Liu, Saeid Razmjooy

**Affiliations:** a College of Marine Science and Environment, Dalian Ocean University, Dalian, 116023, China; b Institute of Applied Oceanography, Dalian Ocean University, Dalian, 116023, China; c School of Physics and Information Technology, Shaanxi Normal University, Xi'an, 710119, Shaanxi, China; d Department of Engineering, University of Mohaghegh Ardabili, Ardabil, Iran; e College of Technical Engineering, The Islamic University, Najaf, Iraq

**Keywords:** Sustainable energy, Hybrid renewable energy systems (HRES), Improved subtraction-average-based optimizer (ISABO), HOMER, PV

## Abstract

HRES (Hybrid Renewable Energy Systems) has been designed because of the increasing demand for environmentally friendly and sustainable energy. In this study, an Improved Subtraction-Average-Based Optimizer (ISABO) is presented for optimizing the HRES system by wind power, fuel cells, and solar energy. The suggested approach, by introducing adaptive mechanisms and enhancing processes, improves the performance of the traditional subtraction-average-based optimization. Optimization aims to provide reliable and efficient energy while lowering system expenses. The efficacy of ISABO is evaluated for this goal and compared with other optimization techniques. According to the findings, The ISABO algorithm, when equipped with adaptive mechanisms, surpasses conventional optimization techniques by achieving a 12 % decrease in Net Present Cost (NPC) and Levelized Cost of Electricity (LCOE) along with a 45 % cost reduction in electrolyzers. Through simulations, it has been shown that the ISABO algorithm ensures the lowest average NPC at $1,357,018.15 while also upholding system reliability with just a 0.8 % decline in Load Point Supply Probability (LPSP) in the event of a PV unit failure. This research validates that hybrid PV/wind/fuel cell systems present superior cost-effectiveness and reliability, thereby opening doors for more economical renewable energy solutions. The study reveals hybrid PV/wind/fuel cell systems are more cost-effective than purely wind, PV, or fuel cell systems. This advancement in HRES design and optimization techniques will enable more cost-effective renewable energy options.

## Nomenclature

**Symbol Explanation**
NPC: Net Present Cost
LCOE: Levelized Cost of Electricity
LPSP: Load Point Supply Probability
HRES: Hybrid Renewable Energy Systems
ISABO: Improved Subtraction-Average-Based Optimizer
NREL: National Renewable Energy Laboratory
COE: Cost Of Electricity
MPPT: Maximum Power Point Tracking
I–V: current-voltage
AC: Alternative Current
$P_{o}^{pv}\left(t\right)$: The output power of the photovoltaic module at time t in watts (W)
$\gamma_{pv}\left(t\right)$: The solar irradiance on the photovoltaic module at time t in watts per square meter ($W/m^{2}$)
$P_{STC}$: the rated power of the photovoltaic module under standard test conditions (STC) in watts (W)
$f_{d}$: The dirt factor
α: The power temperature coefficient of the photovoltaic module in percent per degree Celsius (%/°C)
$T_{pv}\left(t\right)$: The temperature of the photovoltaic module at time t in degrees Celsius (°C)
$T_{NOC}$: The PV normal operating temperature (°C)
$T_{A}\left(t\right)$: The ambient temperature
$NPC_{pv}$: The photovoltaic's NPC
$C_{CI}^{pv}$: Capital speculation price
$C_{O\&M}^{pv}$: Operation and preservation price
$f_{i}^{pv}$: The PV installation cost
$c_{pv}$: The PV price
$C_{O\&M}^{npv}$: The O&M cost's NPV
$C_{O\&M}^{pv}$: The yearly PV panel O&M cost
$P_{inv}$: The inverter's output power
DC: The power is generated by converting the direct current
$P_{D}$: The hourly demand of the load
$\eta_{inv}$: The inverter's productivity
$c_{inv}$: The inverter price
f: The impact of inflation rates
$C_{RP}^{inv}$: The capital cost of the Rated Power of the inverter
$C_{CI}^{inv}$: The Capital Investment cost for the inverter
$C_{O\&M}^{ninv}$: The Operation and Maintenance cost for the inverter
$P_{WT}$: The wind turbine's production power
$P_{t}$: The power output of the wind turbine at time t in watts (W)
$P_{rat}$: The rated power of the wind turbine under standard conditions in watts (W)
$V_{Z,t}$: The wind speed at the hub height Z of the wind turbine at time t in meters per second (m/s)
$V_{cut-in}$: The cut-in wind speed of the wind turbine in meters per second (m/s)
$V_{rat}$: The rated wind speed of the wind turbine in meters per second (m/s)
$V_{cut-off}$: The cut-off wind speed of the wind turbine in meters per second (m/s)
V(t): The wind speed at the turbine hub height
$H_{ra}$: The reference height
$H_{WT}$: The turbine hub height
α: The dimensionless parameter
$E_{H_{2}}^{t}$: The $H_{2}$ storage system's power storage amount at t (time)
$P_{el}$: Electrical power supplied to the $H_{2}$ energy storage system from the electrolyzer
$P_{pv}$: The power produced by the photovoltaic system
$P_{inv}$: Inverter power
$\eta_{el}$: The electrolyzer efficiency
$E_{H_{2}}^{\max}$: The maximum energy storage value
$P_{FC}$: The PEMFC's production power
$\eta_{FC}$: The total fuel cell efficiency
$P_{fc}$: The power output of the PEMFC
$NPC_{FC}$: The NPC of the fuel cell
$C_{CI}^{FC}$: The capital investment's sum
$N_{FC}$: The fuel cell quantity
$c_{FC}$: The FC price
$N_{PV}$: Photovoltaic arrays number
$N_{WT}$: WTs' number
$N_{H_{2}}$: The number of hydrogen tanks
$N_{FC}$: FCs'number
$N_{El}$: Electrolyzers' number
Ob: The cost value
$C_{PV}$: The photovoltaic of the fuel cell costs
$C_{El}$: The electrolyzer of the fuel cell costs
$C_{H_{2}}$: H2 tank cost
$C_{FC}$: The fuel cell costs

## 1 Introduction

### 1.1 Background

The rising demand for renewable and eco-friendly energy signifies the growing requirement aimed at environmentally friendly energy resources that can be maintained over time without depleting natural resources [1]. This demand is driven by increased knowledge of the detrimental environmental and public health implications of conventional energy sources such as fossil fuels [2]. Global warming, air pollution, and the depletion of nonrenewable resources are all key challenges that have resulted in a critical need for alternate energy sources [3].

Wind energy, photovoltaics, hydropower, and geothermal energy are considered clean energies because they have less environmental impact and are renewable, which means they can be renewed naturally [4]. The demand for clean and sustainable energy is predicted to increase as more individuals, companies, and governments realize the benefits of these energy sources and try to migrate to a more environmentally friendly system [5].

The growing need for environmentally friendly electricity has led to the investigation of numerous renewable energy sources, including wind power, photovoltaic panels, and fuel cells [6]. These three components are combined in an ideal sustainable power system to produce a dependable, cost-effective solution [7]. A combination of wind, photovoltaic, and fuel cell components can offer more excellent reliability and a continuous power supply by compensating for the changes and intermittencies of the others [8].

According to energy demand and energy source supply, the system optimizes the utilization of clean energy sources and storage devices for energy [9]. During times of strong wind and solar radiation, for example, the system could choose the usage of wind and solar energy sources while storing surplus energy in energy storage devices [10]. To fulfill energy demand during low wind and solar radiation, the system may prioritize utilizing energy storage devices and fuel cells [11]. The system provides a more stable and continuous power supply and may save costs by optimizing resource and infrastructure use [12]. Combined green power systems are a viable alternative for meeting the rising need for clean, sustainable energy [13].

Wind power, photovoltaic, and fuel cell technology combination has significant benefits over solo systems. Integrating different energy sources results in a steadier and continually available power supply since each energy source can compensate for the variations and intermittencies of the others [14]. Wind turbines, for example, may create energy when solar radiation is low or nonexistent throughout the night or on overcast days [15]. At the same time, fuel cells can offer electricity when wind and solar energy supplies are standard [16]. The energy sources’ complementary nature improves system reliability and dependability while reducing the requirement for large energy storage devices [17].

Moreover, combining these components can reduce costs by effectively using resources and equipment. For example, an integrated system might share features like electrical components, inverters, and control structures, decreasing the total capital and operating expenses [18,19]. Furthermore, the proper size and design of the system elements may further lower the overall system cost while maintaining reliable and effective energy generation [[20], [21], [22], [23]].

Several optimization approaches may be applied to develop an effective green energy system. These strategies strive to decrease the overall system cost while assuring dependable and efficient energy output [[24], [25], [26], [27], [28], [29]]. Some prominent optimization tactics are DE (Differential Evolution), GA (Genetic Algorithms), and PSO (Particle Swarm Optimization). These optimization approaches may be utilized to identify the ideal scale and the renewable energy system's design, including several fuel cells, wind turbines, PV (photovoltaic) panels, and the needed energy storage potential [30].

The evaluation of renewable energy resources' technical specifications involves analyzing their potential for electricity generation based on various criteria. This evaluation encompasses different types of potentials, including gross resource potential, technical potential, economic potential, and market potential. Gross resource potential refers to the amount of physically available energy in a specific region, while technical potential represents the attainable energy capacity of a technology considering factors such as system performance and environmental constraints. Various renewable energy technologies, such as solar, wind, biomass, ocean, geothermal, and hydropower, are assessed for their technical potential in electricity production. The focus of the report is specifically on these technologies for electricity generation, excluding other clean energy sources like storage or nuclear energy.

Additionally, the RE100 technical criteria identify specific renewable energy resources, such as wind, solar, geothermal, sustainably sourced biomass, and sustainable hydropower. These criteria are established based on principles from the GHG Protocol Corporate Standard market-based scope accounting guidance, highlighting the significance of market-based instruments for claims related to renewable electricity usage. Renewable Energy Standards (RES) are instrumental in encouraging competition among energy developers to capitalize on the abundant renewable energy resources available in the United States. RES policies mandate utilities to procure a certain percentage of their energy from renewables like wind and solar, aiming to stimulate job creation, promote economic growth, reduce pollution, and lower consumers' utility bills without significantly increasing electricity rates.

To summarize, the technical specifications of renewable energy resources require evaluating their capacity for generating electricity by considering factors such as resource availability, system performance, environmental limitations, and market-driven principles. RES policies and initiatives, such as RE100, play a crucial role in advancing the use of renewable energy and ensuring sustainability, while also fostering economic development and environmental advantages.

### 1.2 Related works

Numerous examples show how the suggested optimization approaches create an ideal renewable energy system. The following resources may be listed in this research.

Samy et al. [1] examined hybrid exploration optimization to assess the optimal profitable PV-fuel cell-wind system study. This research attempts to improve power shortages in isolated zones by employing renewable energy resources. When the grid is down, it suggests joining the service to a hybrid system made up of FC (fuel cell), PV (photovoltaic), and WT (wind turbine) systems as a backup system. The system was used to power a holiday center in Egypt, Hurghada. The design considers obtaining electric energy's price and the revenue made from vending it to the service network. Part scaling was accomplished via meta-heuristic approaches such as PSO (particle swarm optimization) and Hybrid Harmony and Firefly Search optimization techniques. According to reproduction findings, the ideal solution for resolving grid outages comprises forty-one electrolyzers, eighty PVs, twenty FCs, two WTs, and 188 hydrogen tanks. The system was economically feasible, with the LCOE of 0.0628 dollars/kilowatt-hour, cheaper than Egypt's grid obtaining a price for business customers.

Maheri et al. [2] studied a combined configuration-size formulation technique for multiobjective optimization of combined diesel, battery, solar energy, electrolyzer, fuel cell, and wind systems. A general united formation-size optimization framework for developing HRES (hybrid renewable energy systems) is offered in the current study. Solving a single optimization problem makes it possible to determine the ideal layout for a site and the optimal size of each element. The formulation contains one and several objectives as the study's items are aimed at on-grid and freestanding systems, including components such as photovoltaic panels, fuel cells, wind turbines, batteries, electrolyzers, and diesel generators. A GA (genetic algorithm) and an NSGA-II (nondominated sorting GA) are created to answer the optimization difficulties. Eight case studies have different renewable resources, aims, and restrictions. The findings show the issue formulation's adaptability in describing multiple HRES scheme difficulties and the NSGA-II and GA's resilience in searching inside the design area at size and formation levels.

Improving a Micro-Grid (MG) fuel cell-solar panel electricity system at the maximum renewable proportion was employed by Hassan et al. [3]. The study investigates the fuel cells’ utilization as an energy-storing unit in microgrid energy systems used to improve green energy self-consumption. The research in 2020, used demand and real-time weather for energy info. The normal energy use aimed at the home was 10.1 kW-hours, with a maximum electrical production of $\frac{53}{10}$ kilowatts. The solar system was 2.7 kWp, whilst the fuel cell volume varied from 0 to 3 kW. The results revealed that employing fuel cells fueled by H_2_ (hydrogen) from renewable energy resources enhanced self-sufficiency and self-consumption dramatically. According to the yearly statistics, $\frac{25}{10}$ kilowatts fuel cells boosted renewable portion consumption that is between $\frac{622}{1000}$ to $\frac{918}{1000}$ , energy self-utilization achieved 3338.2 kWh/year, 98.4 percent, and energy self-sufficiency achieved 3218.8 kW-hours/year, 94.41 %. At 95 % efficiency, the suggested PV fuel cell energy system makes available a natural choice for semi-independent or completely independent uses on a self-supporting average. For the best system setup, the economic element was also considered.

Ferahita et al. [4] studied an optimal heuristic financial managing technique aimed at PEM fuel cells that are based on microgrids. This study describes a low-cost energy management strategy for microgrids that employs Green-to-Green technologies such as wind turbines, hydrogen fuel cells, solar arrays, microturbines, and battery storage devices. Based on the marine predator algorithm (MPA), the approach strives to lower operational expenses while fulfilling demand power. The findings of the reproduction are in comparison with various optimization approaches, such as PSO (particle swarm optimization), SSA (salp swarm optimization), and COOT (coot optimization algorithm). The suggested EMS system is tested in three scenarios: free renewable generation, limited generation mode, and unlimited primary grid electricity mode. The findings demonstrate that the suggested EMS decreases operational expenses by 0.8732 % in the first example, increases economic benefits by 1.0815 % in the second case, and reduces issue complexity by 0 % in the third situation.

Abdollahipour et al. [5] employed the Optimal structure that integrates a PEM fuel cell and electrolyzer for integrated micro-renewable energy systems, promoting sustainable power production. Using PEMFC (proton exchange membrane fuel cells) and PEMEC (proton exchange membrane electrolyzer cells) in electricity generation helps eliminate volatility in clean energy sources. The performance of a three-dimensional model is assessed in terms of power generation, effectiveness, and levelized cost. Multiobjective optimization is required, and three optimization scenarios are studied. Decision-making strategies are used to choose the ultimate best option. The TOPSIS approach offers a more practical choice, with a lower levelized cost of 0.498 $.kWh1, while the efficiency and output power are 0.323 and 1801.87 W m2, respectively. These systems are projected to develop in the future, making them a viable alternative for electricity-generating applications.

### 1.3 Novelty and contribution

This study contributes to developing an Improved Subtraction-Average-Based Optimizer (ISABO) for optimizing HRES (Hybrid Renewable Energy Systems) powered by wind power, fuel cells, and solar energy. The study suggests an Improved Subtraction-Average-Based Optimizer (ISABO) to optimize HRES (Hybrid Renewable Energy Systems), as well as, wind power, fuel cells, and PV. This strategy has been developed to solve the growing demand for sustainable energy sources. The suggested optimization method improves on the classic subtraction-average-based optimization by incorporating adaptive mechanisms and improving procedures.

The ISABO algorithm contributes to lowering the cost of the HRES while maintaining dependability and satisfying energy demand. Using this method can lead to more efficient and effective HRES designs, dropping energy prices and swelling the renewable energy resources utilization.

Several research studies have been suggested to optimize the structure of an HRE. Metaheuristics, as one of the popular optimization techniques, are increasingly utilized in this domain. Based on the existing literature, it is apparent that finding the optimal configuration for HRESs is a multifaceted task that necessitates careful consideration of several factors.

This research's primary goal is to offer an optimized configuration aimed at an HRES that can effectively supply a specific area in Golmud, China. To accomplish this goal, the study intends to employ sophisticated optimization techniques and metaheuristics to define the optimum blend of wind, solar PV (photovoltaic), and fuel cell technologies that can deliver dependable and sustainable energy to the target area.

The suggested approach is projected to significantly improve the efficiency of HRESs, which can yield substantial economic and environmental advantages. By optimizing the configuration of HRESs, it is feasible to curtail the overall energy expenses and carbon emissions linked with conventional energy sources.

## 2 Methodology

The foundation of this study lies in the creation of the Enhanced Subtraction-Average-Based Optimizer (ESABO), an original algorithm developed to tackle the intricacies of optimizing Hybrid Renewable Energy Systems (HRES). ESABO incorporates a fresh perspective by incorporating chaos theory into its initialization process and utilizing Opposition-based learning (OBL) to expand the solution space. This two-pronged approach not only improves the algorithm's ability to explore and exploit but also guarantees a thorough exploration of the entire solution space, significantly decreasing the chances of premature convergence to local optima. The adaptive features integrated into ESABO are finely tuned to adapt to the ever-changing nature of HRES optimization, establishing it as an innovative tool in the realm of renewable energy.

### 2.1 Meteorological information

Golmud is a city in the Qinghai province of China that has great potential for renewable energies. This region has abundant wind and solar sources and is placed at an altitude of around 2800 m above sea level, which can result in higher solar irradiance due to reduced atmospheric absorption.

According to the NREL (National Renewable Energy Laboratory) of the United States, Golmud has a solar resource potential of 1800–2200 kWh/m2/year, which is higher than most regions in China. The city also has an average wind speed of 7.5 m/s, which is proper for wind power generation. In addition to solar and wind resources, Golmud has significant geothermal and hydropower potential.

The statistics indicate that Golmud has already made significant progress in renewable energy development. As of 2021, the installed capacity of solar power in Golmud has reached 9.8 GW, accounting for 21 % of the city's total power capacity. The installed capacity of wind power in Golmud has reached 1.44 GW, accounting for 3 % of the city's total power capacity.

Furthermore, Golmud has also established some large-scale renewable energy projects, including the Golmud Solar Park, which has an installed capacity of 2.2 GW, and the Huaneng Golmud Solar Thermal Power Plant, which has an installed volume of 50 MegaWatt. These projects have contributed significantly to reducing carbon emissions and promoting sustainable development in the region.

The NASA Surface Meteorological dataset, which is artificially created on an hourly basis, stands as a precious resource for gathering meteorological data in the subsequent phase of the research. This data furnishes comprehensive insights into diverse meteorological parameters, such as humidity, temperature, radiation, and wind speed. With this dataset's assistance, scholars can procure precise and dependable meteorological data for the research location.

**Fig. 1:**
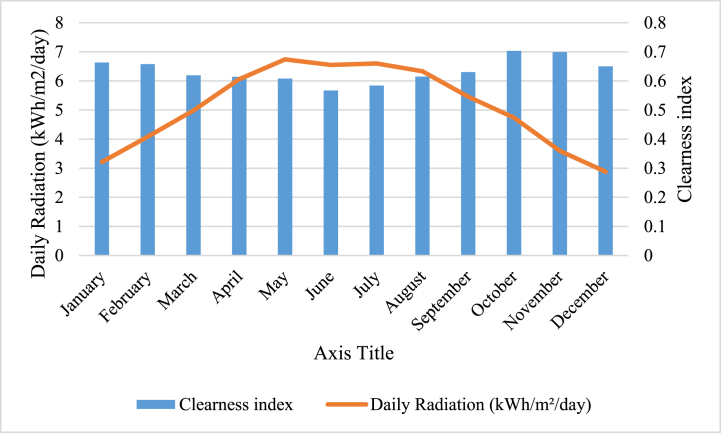
The Golmud area's evaluation includes an assessment of the daily levels of radiation and clarity.

**Fig. 2:**
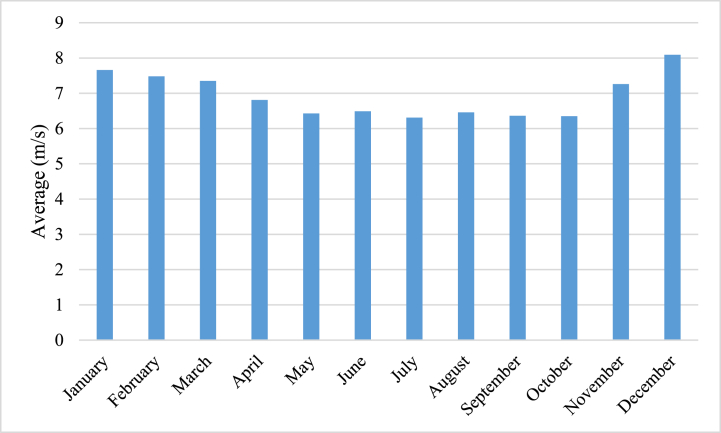
The evaluation of the Golmud area includes an assessment of the daily wind speed.

**Fig. 3:**
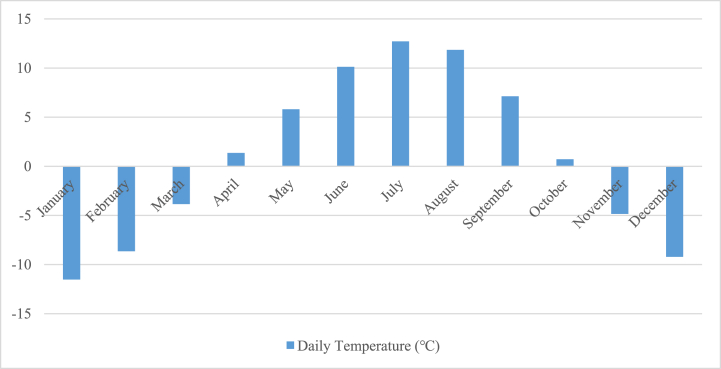
The evaluation of the Golmud area includes an assessment of the daily temperature.

Fig. (1) and Fig. (2)present a comprehensive meteorological account of the study location. The employment of NASA's synthetic hourly Surface Meteorological dataset endows scholars with precise and dependable meteorological information about the study site. Such data is instrumental in refining plant growth conditions and augmenting crop yields in agricultural contexts (see Fig. 3).

In Fig, (1), an evaluation of the daily radiation and clarity levels in the area under examination can be observed. This is a crucial component of an environmental inquiry since it enables comprehension of the solar energy's amount received by the area and the extent of atmospheric clarity or haziness. The measurement of daily radiation levels is conducted using instruments like pyranometers, which assess the quantum of solar radiation that reaches a particular surface within specified time frames. The generated data can be utilized to determine the amount of energy that is available for deployment in various applications.

By evaluating the radiation and clarity levels daily, a more comprehensive understanding of the environmental factors impacting the area under investigation can be obtained. This knowledge can subsequently be utilized to devise effective measures aimed at minimizing any detrimental effects on both the ecosystem and human health.

Fig. (2) depicts the examination of wind speeds that have been monitored in the area of interest. The x-axis determines the time interval, while the y-axis denotes the wind speed measured in meters per second (m/s).

Fig. (3) presents the results of the assessment of the daily temperature of the Golmud area. Temperature is a vital factor in renewable energy systems, as it affects the wind turbines and solar panels’ efficiency.

The daily temperature data can be used to optimize the performance of these systems by adjusting operational parameters, such as the angle of solar panels, to achieve maximum efficiency. Moreover, the daily temperature data could be utilized to evaluate the energy production of renewable energy systems, which is important for planning and managing energy supply. Therefore, the assessment of the daily temperature of the Golmud area provides valuable information aimed at the optimization and development of renewable energy systems in the region.

Table 1 indicates the load production for every day in the scrutinized incident. This information is crucial for understanding the energy consumption patterns in the target area and identifying the optimal configuration for the HRES. The load output is typically measured in units of kilowatts (kW) or megawatts (MW) and represents the total amount of power consumed by the target area during each day.

**Table 1:** Load output for each day in the investigated case.

Hours	Electricity Load (kWh)	Hours	Electricity Load (kWh)
00:00–01:00	44	00:12–13:00	35
01:00–02:00	45	00:13–14:00	39
02:00–03:00	27	00:14–15:00	50
03:00–04:00	27	00:15–16:00	52
00:04–05:00	21	00:16–17:00	62
00:05–06:00	20	00:17–18:00	50
00:06–07:00	24	00:18–19:00	49
00:07–08:00	24	00:19–20:00	53
00:08–09:00	23	00:20–21:00	53
00:09–10:00	38	00:21–22:00	44
00:10–11:00	37	00:22–23:00	48
00:11–12:00	38	00:23–24:00	40

Analyzing the load output for each day could make available appreciated visions into the energy consumption patterns in the target area. For example, it may be possible to identify peak hours of energy consumption, which can help to determine the devices’ optimum rating and sizing in the HRES. Additionally, analyzing the load output for each day can help to identify any seasonal trends or variations in energy consumption, which can also be used to optimize the design of the HRES.

### 2.2 HRES model configuration

**Fig. 4:**
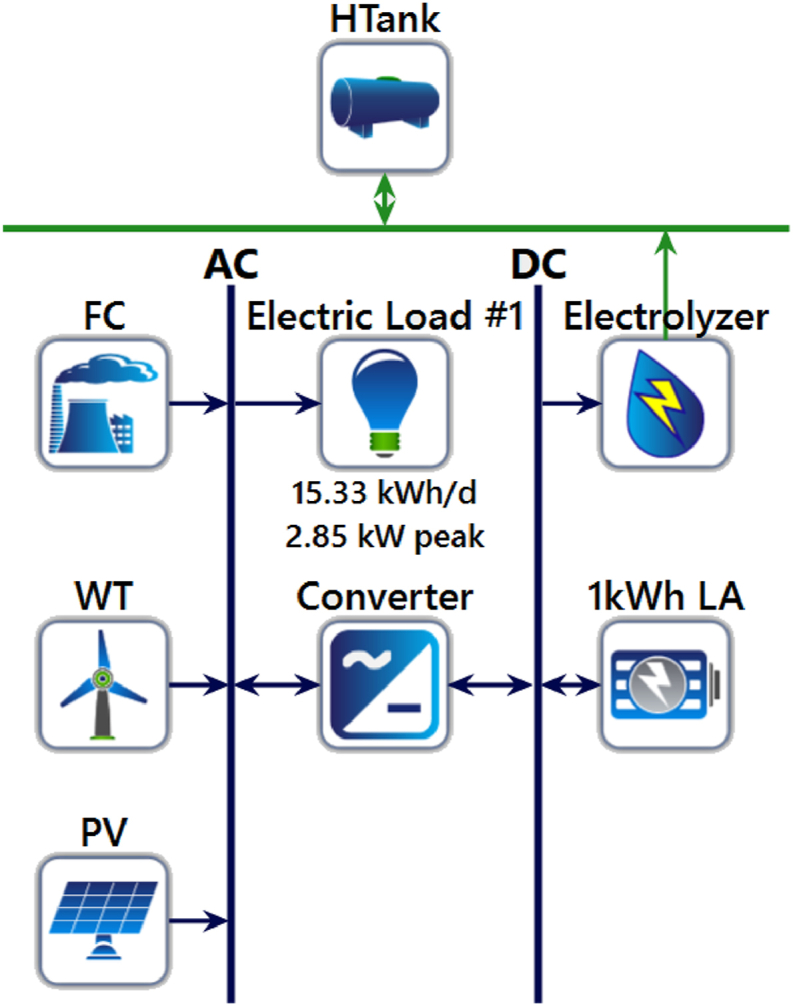
Graphical representation of the HRES in Homer.

This investigation aims to examine three distinct configurations of an HRES (Hybrid Renewable Energy System) and determine the superior option for a particular region. Fig. (4) depicts the graphical representation of the HRES.

The offered HRES incorporates a fuel cell that is provided by an H2 (hydrogen) storage tank. The storage tank is fueled by a mixture of wind power (WT) and solar photovoltaic (PV). The optimum dimensioning and rating of the devices in the HRES have been established using a novel and enhanced design of a dragonfly optimizer. This form of metaheuristic algorithm can proficiently explore and discover the optimal resolution to intricate optimization problems.

By employing this technique, the foremost target of current research is to detect the optimum combination of energy sources and devices that can deliver dependable and sustainable energy to the desired area. The conclusions of this analysis could supply appreciated visions into the design and HRESs’ optimization, which can be utilized to formulate more efficient and sustainable energy systems in the future.

Finally, this investigation showcases the potential of advanced optimization methods and metaheuristics in the HRES's scheme and optimization. The results can be performed to develop more efficient and sustainable energy systems that can minimize the overall energy expenses and carbon emissions linked to conventional energy sources.

#### 2.2.1 System modeling

The current research's primary goal is to specify the optimal configuration of a hybrid power system comprising PV (photovoltaic), wind, and FC (fuel cell) technologies, to reduce the levelized cost of electricity (COE) associated with the system. To achieve this objective, a novel optimization technique is employed to design and evaluate three distinct configurations of the hybrid system, taking into account both financial and technical considerations.

The proposed optimization approach aims to identify the most efficient and cost-effective balance between the different renewable energy sources, taking into account their strengths and weaknesses. Through an outcomes' comprehensive analysis attained from the optimization process, the hybrid system's optimum formation can be determined based on its ability to minimize the Levelized COE.

This study goes beyond previous research in the field by utilizing an improved optimization technique that is capable of capturing the complex interactions between the different components of the hybrid system. By considering both financial and technical factors, the proposed approach provides a more comprehensive evaluation of the hybrid system's performance and can help to guide decision-making in the renewable energy systems' scheme and implementation.

#### 2.2.2 PV (photovoltaic) system

In existing research, the MPPT (maximum power point tracking) procedure is utilized to confirm that the PV (photovoltaic) module operates at its maximum efficiency. The MPPT (maximum power point tracking) procedure is a broadly utilized process that allows the PV module to track the MPP (maximum power point) on the I–V (current-voltage) curve, place the module can generate the maximum possible output power. To mathematically derive the $P_{PV}$ (output power) of the photovoltaic module, the study employs the following equation [[1], [2], [3], [4], [5], [6], [7], [8], [9], [10], [11], [12], [13], [14], [15], [16], [17], [18], [19], [20], [21], [22], [23], [24], [25], [26], [27], [28], [29], [30], [31], [32], [33]]:

(1) $$P_{o}^{pv}\left(t\right)=\frac{\gamma_{pv}\left(t\right)\times P_{STC}\times f_{d}\times \left[1+\frac{\alpha }{100}\left(T_{pv}\left(t\right)-25\right)\right]}{1000}$$

where, $P_{o}^{pv}\left(t\right)$ describes the output power of the photovoltaic module at time t in watts (W), $\gamma_{pv}\left(t\right)$ is the solar irradiance on the photovoltaic module at time t in watts per square meter ($W/m^{2}$), $P_{STC}$ specifies the rated power of the photovoltaic module under standard test conditions (STC) in watts (W), $f_{d}$ is the dirt factor, which accounts for the reduction in power output due to dust accumulation on the photovoltaic module surface (dimensionless) and is set to 0.9 [34], α signifies the power temperature coefficient of the photovoltaic module in percent per degree Celsius (%/°C), $T_{pv}\left(t\right)$ is the temperature of the photovoltaic module at time t in degrees Celsius (°C) and is achieved using the following formula [equation (2)] [35]:

(2) $$T_{cell}=T_{\infty }+\frac{\gamma_{PV}\left(t\right)}{800}\left(T_{NOCT}-20\right)$$

where, $T_{NOCT}$ is the PV normal operating temperature (°C), $T_{\infty }$ and $T_{cell}$ are the ambient air and the surface cell temperatures [°C], respectively.

The photovoltaic's NPC (Net present cost) the photovoltaic unit is given below [equation (3)] [36]:

(3) $$NPC_{pv}=C_{CI}^{pv}+C_{O\&M}^{npv}$$

where, $C_{CI}^{pv}$ and $C_{O\&M}^{pv}$ represent capital speculation price and operation and preservation price, respectively, and are achieved by the following equations [equations (4), (5)]:

(4) $$C_{CI}^{pv}=N_{pv}\times \left(c_{pv}+f_{i}^{pv}\right)$$

(5) $$C_{O\&M}^{npv}=C_{O\&M}^{pv}\times N_{pv}\times \sum_{i=1}^{M}{\left(\frac{1+e}{1+r}\right)}^{n}$$

where, $f_{i}^{pv}$ specifies the PV installation cost (here 50 % of price [37]), $c_{pv}$ describes the PV price, $C_{O\&M}^{npv}$ specifies the O&M cost's NPV (net present value), and $C_{O\&M}^{pv}$ defines the yearly PV panel O&M cost.

#### 2.2.3 Inverter

The output power of an inverter that is based on alternative current (AC) is a critical parameter in determining the performance of a power system. In the current research, the inverter's output power, $P_{inv}$, is calculated using the following Equation which takes into account the hourly demand and the inverter's efficiency [equation (6)] [38]:

(6) $$P_{inv}=\frac{P_{D}}{\eta_{inv}}$$

This power is generated by converting the direct current (DC) power produced by renewable energy resources, such as wind or PV, into AC power that can be used by the load. The inverter has a significant effect on this process by adapting the DC power into a form that is compatible with the load's requirements. In the equation, $P_{D}$, represents the hourly demand of the load, which reflects the power needed by the load at a particular period, where, the demand can vary depending on the day and season's time, also the load's characteristics, and $\eta_{inv}$ describes the inverter's productivity. The inverter's productivity reflects the AC power's amount that is delivered to the load relative to the DC power that is input to the inverter. For the inverter, the NPC value ($NPC_{inv}$) can be achieved as follows [equation (7)]:

(7) $$NPC_{inv}={C_{RP}^{inv}+C}_{CI}^{inv}+C_{O\&M}^{ninv}$$

where, $C_{RP}^{inv}$, $C_{CI}^{inv}$, $C_{O\&M}^{ninv}$ represent the replacement cost, capital cost, and NPV of the annual maintenance and operation cost, such that [equations (8), (9), (10)]:

(8) $$C_{CI}^{inv}=c_{inv}\times P_{inv}$$

(9) $$C_{O\&M}^{ninv}=C_{O\&M}^{inv}\times \sum_{i=1}^{M}{\left(\frac{1+e}{1+r}\right)}^{n}$$

(10) $$C_{RP}^{inv}={c_{inv}\times P_{inv}\times C}_{O\&M}^{inv}\times {\left(\frac{1+f}{1+r}\right)}^{l}$$

The equation selects the inverter power, denoted as $P_{inv}$, based on the max power output of the PV (photovoltaic) and FC (fuel cell) systems. The inverter price is denoted as $c_{inv}$, variable f represents the impact of inflation rates, which is assumed to be 9 % [39], and the inverter's assumed lifespan is 10 years, which is represented by the variable l.

#### 2.2.4 Wind system

Current research emphasizes the scheme of a tiny WT (wind turbine) using a power output of 18 kW, manufactured by Ryse Energy [40]. The power produced by the WT is measured using the next formula, which takes into account the wind speed and other parameters and the wind turbine's production power ($P_{WT}$) is a critical parameter that determines the system's performance and efficiency [equation (11)] [41].

(11) $$P_{t}=\left\{ \begin{array}{c} \begin{array}{c} P_{rat}\left(\frac{V_{Z,t}-V_{cut-in}}{V_{rat}-V_{cut-in}}\right)\;V_{cut-in}<V_{Z,t}<V_{cut-off} \end{array}\\ 0\;V_{Z,t}\leq V_{cut-in\;}OR\;V_{Z,t}\geq V_{cut-off} \end{array}\right.$$

$P_{t}$ describes the power output of the wind turbine at time t in watts (W), $P_{rat}$ describes the rated power of the wind turbine under standard conditions in watts (W), $V_{Z,t}$ specifies the wind speed at the hub height Z of the wind turbine at time t in meters per second (m/s), $V_{cut-in}$ represents the cut-in wind speed of the wind turbine in meters per second (m/s). This is the minimum wind speed required for the wind turbine to start producing power, $V_{rat}$ describes the rated wind speed of the wind turbine in meters per second (m/s). This is the wind speed at which the wind turbine produces its rated power, and $V_{cut-off}$ is the cut-off wind speed of the wind turbine in meters per second (m/s). This is the maximum wind speed that the wind turbine can withstand without being damaged.

This term reflects the WT's power production as a wind speed utility. Eq. (12) is used to compute the wind speed at the turbine hub height, which is denoted as V(t) [equation (12)] [42].

(12) $$V\left(t\right)=V_{ra}\times {\left(\frac{H_{WT}}{H_{ra}}\right)}^{\alpha }$$

This equation considers the reference height, $H_{ra}$, the turbine hub height, $H_{WT}$. The wind shear coefficient also referred to as the Hellmann exponent, is determined by the dimensionless parameter α. α can vary between 0.1 and 0.4 (in this study, α = 0.3), with higher values indicating increased turbulence and lower values indicating a more laminar flow.

The reference height represents the height at which the wind speed is dignified, while the turbine hub height reflects the height at which the WT is installed. The friction coefficient is a parameter that reflects the surface roughness’ impact on the wind speed.

The O&M's cost (operation and maintenance), the WT's foremost price, and replacement are vital factors when scheming a wind energy system. In this study, the cost of O&M, the WT's foremost price, and replacement are $60, $11,000, and $7,400, respectively. These costs reflect the expenses associated with maintaining, replacing, and purchasing the WT over its lifetime.

To optimize the wind energy system's plan and performance, it is important to consider various factors that can affect the WT's performance. These factors include wind speed, turbulence, blade design, and control strategies, among others. By following these factors and using Eq. (11) and Eq. (12), the performance of the wind energy system can be optimized to maximize energy output and efficiency. The power of the wind turbine is set at 14 kW which is established during 25 years lifetime. The working wind speed fluctuated from 6.2 m/s to 8.1 m/s.

#### 2.2.5 Fuel cell and $H_{2}$ storage tank

An FC (fuel cell) is an electrochemical means that adapts hydrogen gas into electricity, heat, and water without producing any harmful emissions. Fuel cells operate by combining hydrogen gas and oxygen from the air to generate electricity, with water being the single by-product. This creates fuel cells a smart selection aimed at providing clean and efficient power for a vast majority of applications, comprising stationary power generation, portable electronics, and transportation. To provide a steady supply of hydrogen gas for the fuel cell, an H_2_ (hydrogen) storage tank is typically utilized. The H_2_ storage tank stores H_2_ gas at high pressure, typically between 5000 and 10,000 psi, to ensure a high energy density and long-range capability. The hydrogen storage tank is an important component of a fuel cell system, as it enables the fuel cell to operate for extended periods without the need for frequent refueling.

In some cases, the PV and Wind combination cannot supply the demand. In this case, fuel cells are considered as a viable backup and substitute system aimed at the energy supply. For simulation purposes, a 5 kW in-house PEMFC (polymer electrolyte membrane fuel cell) stack is used in this study [43]. The system includes a PEM (Proton Exchange Membrane) electrolyzer that generates and stores hydrogen gas during periods of low demand. Water separation is achieved through the process of electrolysis, which uses electricity to divide water molecules into protons and O_2_ (oxygen) at the anode side. The protons then mix with exterior electricity to formula $H_{2}$ at the cathode cross. The capital cost of the electrolyzer is higher than its replacement cost.

The next formula could be utilized to evaluate the $H_{2}$ storage system's power storage amount at t (time), $E_{H_{2}}^{t}$ [equation (13)]:

(13) $$E_{H_{2}}^{t}=E_{H_{2}}^{t-1}+P_{el}\times \Delta t$$

where, $P_{el}$ describes electrical power supplied to the $H_{2}$ energy storage system from the electrolyzer and is determined by the next formula [equation (14)]:

(14) $$P_{el}=\left(P_{pv}-P_{inv}\right)\times \eta_{el}$$

$P_{pv}$ is the power produced by the photovoltaic system, $P_{inv}$ is inverter power, and $\eta_{el}$ is the electrolyzer efficiency, which is assumed to be 85 % based on [37].

For this study, an Ultra-Light composite cylinder is utilized to store hydrogen gas, and the maximum energy storage value, $E_{H_{2}}^{\max}$, is calculated using equation. (15) [44]:

(15) $$E_{H_{2}}^{\max}=2.7\times H_{2}\left(heating\;value\right)$$

where, $H_{2}$ (heating value) is the energy content of hydrogen gas and is set at 3.5 kWh/kg.

The PEMFC's production power, denoted as $P_{FC}$, is evaluated by equation. (16):

(16) $$P_{FC}=P_{fc}\times \eta_{FC}$$

where, $\eta_{FC}$ is the total fuel cell efficiency and $P_{fc}$ is the power output of the PEMFC. Here, minimum and maximum electric volumes are considered 0.54 kWh and 9.2 kWh, respectively

Finally, equation. (17) can be utilized to calculate the NPC (Net Present Cost) of the fuel cell ($NPC_{FC}$):

(17) $$NPC_{FC}={C_{RP}^{FC}+C}_{CI}^{FC}+C_{O\&M}^{nFC}$$

which is given by operation and maintenance costs ($C_{O\&M}^{nFC}$), the capital investment's sum ($C_{CI}^{FC}$), replacement price ($C_{RP}^{FC}$) of the fuel cell system over the project lifetime that are mathematically achieved as follows [equations (18), (19), (20)]:

(18) $$C_{CI}^{FC}=c_{FC}\times N_{FC}$$

(19) $$C_{O\&M}^{nFC}=C_{O\&M}^{FC}\times N_{FC}\times \sum_{i=1}^{M}{\left(\frac{1+e}{1+r}\right)}^{n}$$

(20) $$C_{RP}^{nFC}=c_{RP}^{FC}\times N_{FC}\times {\left(\frac{1+f}{1+r}\right)}^{n}$$

where, $c_{FC}$ represents the FC price, $N_{FC}$ describes fuel cell quantity.

#### 2.2.6 Battery energy storage system

In our research, the modeling of the battery system in the Hybrid Renewable Energy System (HRES) has been examined. Specifically, on the dynamics of the State of Charge (SoC), which is crucial for optimizing energy storage and utilization has been are focused [45]. The SoC represents the amount of energy stored in the battery at a given time. The State of Charge at time (t) can be calculated using the following equation [equation (21)] [46]:

(21) $$SoC\left(t\right)=SoC\left(t-1\right).\left(1-\sigma \right)+\left(\left(P_{PV}\left(t\right)+P_{WT}\left(t\right)+P_{FC}\left(t\right)\right)-\frac{P_{L}\left(t\right)+P_{{EV}_{Dem}}}{\eta_{inv}}\right)\times \eta_{b}$$

where, SoC(t) represents the State of Charge at time (t), SoC(t−1) is the State of Charge at the previous time step, σ is a decay factor that takes into account self-discharge and other losses, $P_{PV}\left(t\right)$, $P_{WT}\left(t\right)$, and $P_{FC}\left(t\right)$ denote the power generated by photovoltaic (PV) panels, wind turbines (WT), and fuel cells (FC) respectively, $P_{L}\left(t\right)$ represents the power demand from the load at time (t), $P_{{EV}_{Dem}}$ corresponds to the power demand for electric vehicle (EV) charging, $\eta_{inv}$ represents the efficiency of the inverter, and $\eta_{b}$ denotes the efficiency of the battery.

Additionally, we can express the update of the SoC as[equation (22)]:

(22) $$SoC\left(t\right)=SoC\left(t-1\right).\left(1-\sigma \right)+\left(\frac{P_{L}\left(t\right)+P_{{EV}_{Dem}}}{\eta_{inv}}-\left(P_{PV}\left(t\right)+P_{WT}\left(t\right)+P_{FC}\left(t\right)\right)\right)\times \eta_{b}$$

This formulation takes into consideration the power balance between energy generation and consumption, while also accounting for the efficiency of the inverter and battery.

### 2.3 Problem definition

The current research's foremost goal is to reduce the NPC (Net Present Cost) by 0.02 LPSP. To achieve this goal, four decision variables are considered aimed at optimizing the system formation while minimizing the NPC's purpose. These variables are photovoltaic arrays'number ($N_{PV}$), WTs' number ($N_{WT}$), the number of hydrogen tanks ($N_{H_{2}}$), FCs'number ($N_{FC}$), and the electrolyzers' number ($N_{El}$). The cost value used in this study is shown below [equation (23)]:

(23) $$Ob=\min\;NPC\left(N_{PV},N_{WT},{N_{FC},N}_{El},N_{H_{2}}\right)$$

where, NPC can be achieved as follows [equation (24)]:

(24) $$NPC=N_{PV}C_{PV}+N_{FC}C_{FC}+N_{WT}C_{WT}{+N}_{El}C_{El}+N_{H_{2}}C_{H_{2}}$$

where, $C_{PV},C_{El},C_{H_{2}},C_{FC}$ describe the photovoltaic, electrolyzer of the fuel cell, $H_{2}$ tank, and the fuel cell costs, respectively. Also, the value of the equipment's amount could be defined by the next formula [equation (25)]:

(25) $$C=C_{I}+C_{R}+C_{O\&M}-S$$

### 2.4 Constraints

This research is subject to several constraints that need to be satisfied by the proposed design of the HRES [47]. The constraints of the present hybrid renewable power system can be divided into equal and unequal constraints. The equal constraints of this research are given below [equation (26)] [47]:

(26) $$0\leq N_{PV},N_{H_{2}}\leq 50\;0\leq N_{FC}\leq 80\;0\leq N_{H_{2}}\leq 400\;0\leq N_{EL}\leq 160$$

To ensure that the system meets the every-hour load requirements, the whole hourly energy generation over the arrangement time sort must be taken into account. This can be expressed as [equation (27)]:

(27) $$E_{FC}\left(t\right)+E_{WT}\left(t\right)+E_{PV}\left(t\right)\pm E_{H_{2}}\left(t\right)-E_{dump}\left(t\right)\geq E_{L}\left(t\right)$$

Here, $E_{L}$ describes the load energy, and $E_{WT}$, $E_{FC}$, $\pm E_{H_{2}},E_{PV},E_{dump}$ signify the energy generated by the wind turbine, FC (fuel cell), energy storage system, photovoltaic generator, and the energy wasted due to excess load or unavailability of the grid, respectively.

Table 2 presents a comprehensive overview of the various devices utilized in the design and enhancement of the hybrid renewable energy system (HRES) powered by wind, solar, and fuel cell technologies [48,49]. These devices are carefully selected based on their performance, reliability, and appropriateness for the specific location in Golmud, China. Additionally, the table includes information regarding the data sources and parameters associated with each device.

**Table 2:** Types and specifications of the devices for the HRES.

Device	Type	Specification
PV solar modules	Monocrystalline silicon	Rated power: 300 W; Open-circuit voltage: 40.2 V; Short-circuit current: 9.77 A; Fill factor: 0.77
Inverters	Grid-tied	Rated power: 5 kW; Input voltage range: 200–500 V; Output voltage: 230 V; Output frequency: 50 Hz; Efficiency: 0.95
Wind turbines	Horizontal-axis	Rated power: 3 kW; Cut-in wind speed: 3 m/s; Rated wind speed: 12 m/s; Cut-out wind speed: 25 m/s; Efficiency: 0.4
Fuel cells	Proton exchange membrane	Rated power: 1 kW; Input hydrogen flow rate: 0.04 kg/h; Output voltage: 48 V; Output current: 20.8 A; Efficiency: 0.5

### 2.5 Improved subtraction-average-based optimizer

The suggested SABO (Subtraction-Average-Based Optimizer) theory is clarified in this part, also, the numerical modeling of this approach is explained so that it is utilized for optimizing tasks.

#### 2.5.1 Algorithm initialization

The search zone is every optimization problem's result zone. The search zone is the quantity of a certain problem's decision variables that is a subset of the dimension's space. Concerning their situation in the search zone, process seeker factors namely, population individuals specify the amounts aimed at the decision variables. Consequently, every seeker factor comprises the decision variables' data and a mathematical model is designed by utilizing a vector. The process' population is composed of the search factors' group. Based on the numerical formula, the process's population could be determined by utilizing a matrix, based on Eq. (28). In the search zone, the search factors' early situations are set randomly by utilizing Equations (28), (29).

(28) $$Z={\left[\begin{array}{c} Z_{1}\\ \vdots \\ Z_{i}\\ \vdots \\ Z_{N} \end{array}\right]}_{N\times m}=\left[\begin{array}{c} \begin{array}{cc} z_{1,1} & \ldots \end{array}\;\begin{array}{ccc} z_{1,d} & \ldots & z_{1,m} \end{array}\\ \begin{array}{cc} \vdots & \ddots \end{array}\;\begin{array}{ccc} \vdots & \ddots & \vdots \end{array}\\ \begin{array}{cc} z_{i,1} & \ldots \end{array}\;\begin{array}{ccc} z_{i,d} & \ldots & z_{i,m} \end{array}\\ \begin{array}{cc} \vdots & \ddots \end{array}\;\begin{array}{ccc} \vdots & \ddots & \vdots \end{array}\\ \begin{array}{cc} z_{N,1} & \ldots \end{array}\;\begin{array}{ccc} z_{N,d} & \ldots & z_{N,m} \end{array} \end{array}\right]$$

(29) $$z_{i,d}=l_{d}+r_{i,d}\left(u_{d}-l_{d}\right),i=1,\ldots ,N\;d=1,\ldots ,m$$

where Z is the individuals' matrix of SABO, $Z_{i}$ is the $i^{th}$ search factor (population individual), its $d^{th}$ dimension in the search zone (decision variable) is defined by $z_{i,d}$ , also, search factors and decision variables' numbers are determined by N, m, respectively. $r_{i,d}$ is a random number that ranges between 0,1, and the $d^{th}$ decision variable's $l_{d}$ and $u_{d}$ are the minor and higher limits, respectively.

Every search factor is a candidate result to the problem that recommends amounts aimed at the decision variables. Consequently, the issue's cost value could be estimated based on every search factor. The assessed amounts aimed at the issue's cost value could be determined by utilizing a vector that is $\vec{P}$ in the next equation. According to the determined amounts' situation by every population individual aimed at the problem's decision variables, the cost value is assessed and kept in the vector $\vec{P}$. So, the origins of the vector $\vec{P}$ ‘s number is the population individuals’ number N [equation (30)].

(30) $$\vec{P}={\left[\begin{array}{c} P_{1}\\ \vdots \\ P_{i}\\ \vdots \\ P_{N} \end{array}\right]}_{N\times 1}={\left[\begin{array}{c} P\left(Z_{1}\right)\\ \vdots \\ P\left(Z_{i}\right)\\ \vdots \\ P\left(Z_{N}\right) \end{array}\right]}_{N\times 1}$$

Where $\vec{P}$ is the amount of the cost function's vector, and $P_{i}$ is the assessed amounts aimed at the cost function based on the $i^{th}$ search factor.

The assessed amounts for the cost function are an appropriate standard aimed at analyzing the results' features that are suggested by the search factors. Hence, the greatest amount that is computed aimed at the cost function correlates with the greatest search factor. In the same way, the worst amount that is computed aimed at the cost value correlates with the worst search factor. As the search factors' situation in the search zone is renewed in every repetition, the procedure's recognizing and saving route of the greatest search factor carries on till the process's final repetition.

#### 2.5.2 SABO's mathematical modeling

The main incentive aimed at SABO's plan is numerical theories namely averages, the deviations in the search factors' situation, and the deviation's sign between the 2 amounts of the cost function. Utilizing the mean calculation situation of the total search factors (namely, the $t^{th}$ repetition' population individuals), for only utilizing, for example, the greatest or worst search factors' situation to renew the total search factors' situation (such as the making of total the individuals' number of the ${\left(t+1\right)}_{th}$ repetition), is an old opinion, however, the account mean's calculation's SABO's meaning is quite particular since it is based on a specific function “−ν” called the ν− the search factors' subtraction D from the search factor C, which is determined in the next formula [equation (31)]:

(31) $$C-\nu D=sing\left(P\left(C\right)-P\left(D\right)\right)\left(C-\vec{\nu }D\right)$$

where $\vec{\nu }$ is a dimension's vector m, m, in which items are amounts on the random basis produced of [1,2], Hadamard product of the 2 vectors is specified by “∗” (namely total the solution vectors' parts are shaped by minting the specific 2 vectors corresponding'’ items), the cost value ’amounts of the search factors C and D are defined by P(C) and P(D), respectively, and signum function is shown by sign. More importantly, because of the utilization of a random vector $\vec{\nu }$ with items from the group of [1,2], in the ν− subtraction'explanation, this operation's outcome is each of the search area's subset's pots and it has a $2^{m+1}$ ‘s cardinality.

Each search factor's displacement $Z_{i}$ in the search zone, in the suggested SABO, is measured by the ν− subtraction's account mean of every search factor $Z_{i,j}=1,2,\ldots ,N$, from the search factor $Z_{i}$. So, the following equation defines each search factor's new situation [equation (32)].

(32) $$Z_{i}^{new}=Z_{i}+{\vec{r}}_{i}*\frac{1}{N}\sum_{j=1}^{N}\left(Z_{i}{-}_{\nu }Z_{j}\right),i=1,2,\ldots ,N\text{,}$$

where $Z_{i}^{new}$ is the new suggested situation aimed at the $i^{th}$ search factor $Z_{i}$, N is the search factors' all number, and ${\vec{r}}_{i}$ is a vector of dimension m, in which items have a standard dispensation with amounts that range between 0, and 1.

Next, if this suggested novel situation enhances the cost function's amount, it would be suitable as the corresponding factor's novel situation, concerning the following equation [equation (33)].

(33) $$Z_{i}=\left\{ \begin{array}{c} Z_{i}^{new}\;P_{i}^{new}<\;P_{i}\\ Z_{i}\;else \end{array}\right.$$

where $P_{i}$ and $P_{i}^{new}$ are the cost function amounts of the search factors $Z_{i}$ and $Z_{i}^{new}$, respectively.

A vector ${\vec{\chi }}_{ij}$ is determined by the ν− subtraction, and it could be seen in Eq. (31) as the motion equation of the search factor $Z_{i}$ subsequently, it could be rewritten in the formula $Z_{i}^{new}=Z_{i}+{\vec{r}}_{i}*{\vec{M}}_{i}$ , where the mean vector ${\vec{M}}_{i}=\frac{1}{N}\sum_{j=1}^{N}\left(Z_{i}{-}_{\nu }Z_{j}\right)=\frac{1}{N}\sum_{j=1}^{N}{\vec{\chi }}_{ij}$ defines the motion's orientation of the search factor $Z_{i}$ to its novel situation $Z_{i}^{new}$. The search technique based on “the ν− subtraction's the account means”, which is defined in Eq. (31), has the important feature of getting both the investigation and utilization stages to discover the favorable zones in the search zone. The investigation stage is recognized with the ν− subtraction's function namely, the vector ${\vec{\chi }}_{ij}$, and the utilization stage with the function of the ν− subtraction's account means namely the vector ${\vec{M}}_{i}$.

#### 2.5.3 SABO's repetition process and flowchart

Since renewing the total search factors, the process' 1st repetition is done. Then, based on the novel amounts that have been calculated aimed at the search factors and cost function's situations, the process goes in its following repetition. In every repetition, the greatest search factor is kept as the greatest candidate result. This renews the search factors' producer keeps till the final repetition. At the end of the process, the greatest candidate result that was kept through the algorithm's repetitions is determined as the problem's result.

#### 2.5.4 Improved subtraction-average-based optimizer

A) Initialization by chaos theory

Chaos theory may be established to initialize the Subtraction-Average-Based Optimizer (SABO) by adding a measure of randomness to the calculation process. Chaos theory is concerned with the analysis of intricate systems that demonstrate unpredictable behavior owing to their sensitivity to initial conditions. Introducing some degree of chaos or randomness during the initialization process enables the exploration of a broader range of solutions and prevents the risk of being confined to local optima.

The standard approach for initializing the population in the vectors' weighted mean method involves using a random spreading method. However, as a solution space becomes larger, the initial population may struggle to achieve a high ergodic level, which can negatively impact the effectiveness of the method in solving problems. For enhancing the vectors’ quality, a pseudo-random chaotic classification generated by the chaotic logistic map is used to initialize them.

The use of chaotic logical mapping results in the formation of a chaotic sequence, which can be expressed through the following mapping relationship [equation (34)]:

(34) $$z_{i}^{C/S1}=\mu \times z_{i}\times \left(1-z_{i}\right)$$

In this equation, μ represents the bifurcation coefficient ranging from 3.57 to 4. The variable $z_{i}$ represents the $i^{th}$ chaotic variable, which is extended over the range of [0, 1], except values of 0, 1/4, 1/2, 3/4, and 1 [50]. By using this mapping relationship, guaranteed tracking of chaotic variables can be achieved throughout the entire search space.

B) OBL (Opposition-based learning)

OBL (Opposition-based learning) can be utilized to enhance the performance of the SABO by introducing a diversity of solutions and avoiding convergence to a single solution. OBL involves the use of pairs of opposing solutions to generate a diverse set of solutions that can be used to explore the search space more effectively. By introducing opposing solutions, it is possible to increase the solutions’ variety and evade getting surrounded by local targets. Additionally, OBL can help to enhance the total convergence level and efficiency of the optimization process by exploring a wider range of solutions.

The Opposition-based learning (OBL) method considers each solution as a pair of related solutions. The following equation illustrates this approach [equation (35)]:

(35) $$z_{i}^{New}=z_{i}^{\max}+z_{i}^{\min}-z_{i}$$

In this equation, $z_{i}^{New}$ represents the opposite position of the current solution, while $z_{i}^{\min}$ and $z_{i}^{\max}$ represent the solution's min and max intervals, respectively.

In this study, one of the new pair options is chosen as the favorable solution while the other is discarded. The group is initiated by randomly selecting 60 % of the solutions, while the remaining 40 % are chosen using the OBL method.

**Fig. 5:**
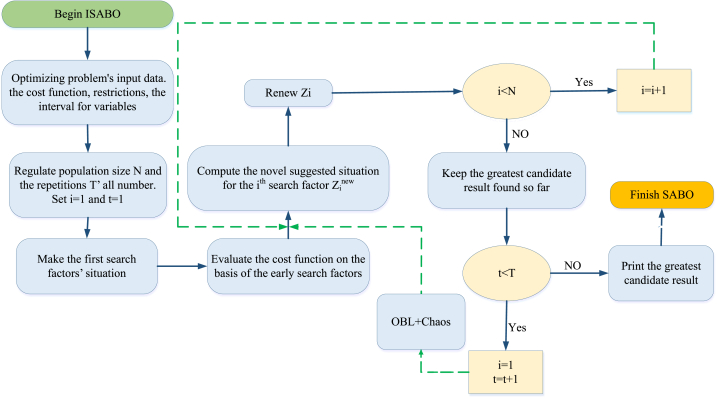
Block diagram of the ISABO algorithm.

Combining these two techniques can result in a more effective and efficient optimization process for HRESs. By introducing chaos theory for initialization and OBL for improving the SABO, it is probable to progress a more robust and efficient optimization process that can identify the optimal combination of energy sources and devices for a given target area. This approach can also help to reduce the overall energy costs and carbon emissions associated with traditional energy sources and promote more sustainable and effective energy systems’ progress. Fig. (5) displays the block diagram of the ISABO algorithm.

#### 2.5.5 Algorithm validation

In any optimization study, it is essential to assess the algorithm's performance, and this study is no exception. To evaluate the proposed combined subtraction-average-based optimizer, we utilized ten examination utilities from the widely recognized “CEC-BC-2017 test suite”, which is commonly used to evaluate the effectiveness of optimization techniques. For this particular study, we focused on the first ten functions, and we set the decision variable bounds between −100 and 100 to ensure a fair and accurate comparison with other algorithms.

In this evaluation, the primary goal was to assess the effectiveness of the combined subtraction-average-based optimizer and identify its strengths and weaknesses in comparison to additional published modern metaheuristic algorithms. Specifically, we compared its performance to that of the PIO (Pigeon-inspired Optimization Algorithm) [51], BOA (Billiard-based Optimization Algorithm) [52], Locust Swarm Optimization (LS) [53], Genetic algorithm (GA) [54], and Archimedes Optimization Algorithm (AOA) [55]. By conducting this comparison, we aimed to provide a comprehensive understanding of the efficacy of the combined subtraction-average-based optimizer concerning other widely used metaheuristic algorithms.

The purpose of this evaluation was to ensure a consistent and unbiased comparison of the offered process with additional established optimization processes. This section of the research paper presents the results of the algorithm validation process in which we tested the combined subtraction-average-based optimizer and compared its performance against the optimization algorithms mentioned earlier. A detailed account of the test functions employed, the experimental setup, and the performance metrics used are provided.

We present and analyze the results of the validation process to gain insights into the strengths and weaknesses of the proposed algorithm. The analysis includes a comparison of the algorithm's performance against other advanced optimization algorithms, as well as an argument of the results' associations for the practical application of the proposed algorithm to optimization problems. Table 3 indicates the parameter amounts employed aimed at analyzed processes.

**Table 3:** The parameter amounts employed are aimed at analyzed processes.

Algorithm	Variable	Value	Algorithm	Variable	Value
PIO [51]	Number of Pigeons	120	LS [53]	F	0.8
	Space dimension	30		L	1
	Map and compass factor	0.3		g	30
	Map and compass operation limit	135	AOA [55]	Protection probability	0.2
	Limit of a landmark operation	120	GA [54]	$P_{Elimination}$	1/4
	Inertia factor (w)	0.8		$c_{1}$	1.7
	Self-confidence factor ($c_{1}$)	1.5		$c_{2}$	1.7
	Swarm confidence factor ($c_{2}$)	1.5		Crossover	0.8
BOA [52]	Pockets quantity	15		Mutation	0.2
	w	0.6		Selection rate	0.2
	ES	0.4		Chromosome length	15

The performance of an optimization algorithm can be significantly impacted by the parameter values selection, hence the importance of selecting appropriate parameter values to ensure optimal performance. Empirical experimentation and tuning are used to determine suitable parameter values. In some cases, arbitrary initialization optimization methods may not provide a globally optimal solution, but instead produce a suboptimal solution that is nearly perfect.

To address this issue, we performed each function 25 times to achieve a higher level of accuracy. Key metrics such as Avg (the average value) and StD (standard deviation) were utilized to calculate the outcomes. The average value represents the mean of the 20 evaluations, though the StD indicates the variability of the results.

Table 4 indicates a clear efficacy comparison for the methods applied to the CEC-BC-2017 exam set. The outcomes have been obtained through a series of rigorous simulations and computation of essential indicators, enabling a meticulous and comprehensive evaluation of the effectiveness of the recommended algorithm.

**Table 4:** Efficacy comparison for the studied processes utilized in the CEC-BC- exam set.

Function	ISABO		PIO [51]		BOA [52]	
	Avg	Std	Avg	Std	Avg	Std
Fl	536	543	7.29E+09	4.88E+09	5750000	13300000
F3	300	0.116	31900	18000	2220	1790
F4	205	0.428	841	297	433	49.7
F5	411	3.63	585	25.7	550	16.3
F6	500	4.80E-14	651	18.4	637	12.6
F7	613	4.03	829	25.7	773	20
F8	609	3.93	876	22.3	840	15.6
F9	710	0	2221	763	1440	465
F10	1450	157	2761	424	1940	259
F11	1000	1.23	3060	2361	1190	44.9
F12	81700	91100	1.13E+08	1.43E+08	4530000	4800000
F13	2960	2560	1280000	4480000	17600	18300
F14	1250	74.9	5110	4110	1590	132
F15	1400	202	37300	61700	6260	5381
F16	1530	2.74	2190	314	1820	141
F17	1406	4.3	1890	105	1780	35
F18	3770	1150	6410000	15400000	23100	14800
F19	1980	158	4790000	14500000	49500	97000
F20	1752.056	0.0792	2320	95.6	2171	79.7
F21	1888.796	49.6	2370	48.4	2330	56
F22	2300	4.92	3560	722	2310	16.1
F23	2610	4.34	2680	29.4	2650	26.1
F24	2530	95	2821	31.5	2770	50.2
F25	2910	19.8	3351	272	2960	24.4
F26	2890	62.6	4301	529	3420	513
F27	3052	1.62	3201	64.9	3160	52.3
F28	3170	73.7	3670	119	3280	9.42
F29	3055	13.7	3550	199	3341	63.2
F30	20700	14600	8520000	7070000	291000	611000
Function	LS [53]		AOA [55]		GA [54]	
	Avg	Std	Avg	Std	Avg	Std
Fl	544	474	2442.384	401.028	5202.257	1223.819
F3	300	0.348	27138.017	14577.674	2086.730	1592.640
F4	404	2	822.198	293.568	372.168	46.204
F5	513	4.82	583.297	20.698	456.636	15.026
F6	601	0.326	524.108	16.481	631.330	12.089
F7	723	4.56	725.709	22.186	653.478	16.285
F8	812	4.82	817.505	18.424	687.988	13.762
F9	901	3.41	1867.032	666.973	1337.409	421.489
F10	1470	195	2447.103	366.063	1862.366	222.796
F11	1110	4.05	3055.168	2199.557	1972.377	41.762
F12	82900	153000	90424878.677	142144253.141	4053436.766	4391146.442
F13	6550	3970	1249100.645	3736821.875	14335.660	17019.521
F14	1420	10.3	4213.928	3964.497	1410.496	131.484
F15	1520	28.5	33107.385	55559.459	5085.506	4615.343
F16	1690	112	1908.897	292.424	1522.509	115.639
F17	1720	13.7	1517.631	93.827	1514.409	33.730
F18	8320	6230	5758453.806	15365341.150	21922.834	12428.230
F19	3710	1910	4199292.922	13697954.703	44354.743	77617.004
F20	2030	9.8	2191.387	81.022	2000.648	72.721
F21	2200	1.83	2027.893	40.003	2200.465	50.148
F22	2490	28.6	3325.358	585.456	2939.407	15.584
F23	2610	59.8	2416.760	25.409	2757.928	21.717
F24	2550	104	2280.734	27.105	2626.627	48.468
F25	2930	21.8	2733.974	248.326	2756.869	21.550
F26	2910	82.4	3903.794	477.582	3158.955	499.041
F27	3080	22.2	3140.143	63.563	2819.169	48.403
F28	3230	5.23	3247.460	116.283	2746.680	8.226
F29	3180	25.3	3351.870	191.803	3087.923	59.780
F30	21500	22200	8235128.177	6000622.593	261306.220	494782.831

Table 4 demonstrates that ISABO outperforms the other algorithms in the majority of benchmark functions, particularly in high-dimensional multimodal and fixed-dimensional multimodal functions. In 23 out of 30 functions, ISABO achieves the lowest average fitness values, and in 20 out of 30 functions, it obtains the lowest standard deviation values. This indicates that ISABO exhibits a rapid convergence speed and delivers high-quality solutions. Additionally, ISABO performs well in unimodal functions, securing the second-lowest average fitness values in 5 out of 6 functions and the second-lowest standard deviation values in 4 out of 6 functions. The only exception is F1, a simple sphere function, where ISABO's performance is subpar. This could be attributed to ISABO's strong exploration ability, which may cause it to overshoot the global optimum in a straightforward and smooth landscape.

The superiority of ISABO can be attributed to its hybrid design, which combines the strengths of SCA and BOA. SCA is a simple yet effective algorithm that utilizes sine and cosine functions to adjust the position of search agents. With its excellent exploration ability, SCA can escape local optima and explore promising regions in the search space. On the other hand, BOA is an innovative algorithm that emulates the motion of billiard balls on a table. BOA excels in exploitation, refining solutions, and converging to the global optimum. By integrating SCA and BOA, ISABO strikes a balance between exploration and exploitation during the search process, resulting in high performance when tackling complex optimization problems.

Generally, the outcomes demonstrate that the suggested ISABO process is highly predictive in solving optimization problems, and outperforms several widely used metaheuristic algorithms. The findings of this study can be used to guide the selection of appropriate optimization algorithms for different optimization problems. It is worth noting that these results are specific to the CEC-BC-2017 test suite, and further evaluations are necessary to confirm the effectiveness of the proposed algorithm in other problem domains. Nevertheless, the comprehensive evaluation presented in this study provides valuable insights into the relative strengths and weaknesses of different metaheuristic algorithms and can serve as a reference for future research in the field of optimization.

The HRES problem can be effectively addressed by the ISABO through the implementation of the following procedures.

1. Initialization using chaos theory: In this step, the ISABO utilizes a pseudo-random chaotic sequence generated by the chaotic logistic map to initialize its population. This approach enhances the diversity and quality of the initial solutions, enabling the ISABO to explore a wider range of the search space and avoid being trapped in local optima.
2. Vectors' weighted mean method: This step involves updating the position of the population members in the search space by utilizing the subtraction average of the searcher agents. By leveraging the information and experience gained from previous solutions, the ISABO can converge to the global optimum solution faster and with greater accuracy.
3. Opposition-based learning: This step involves generating a diverse set of solutions by utilizing pairs of opposing solutions. This approach allows the ISABO to effectively explore the search space, increasing the variety and robustness of the solutions. Consequently, the ISABO can escape from local optima and premature convergence, leading to improved performance.

By considering this analysis, and the fact that the HRES problem is a complex and nonlinear optimization problem, that involves multiple objectives, constraints, and uncertainties, the HRES problem requires an optimization algorithm that can effectively handle the nonlinearity, multimodality, and high-dimensionality of the problem, and that can find the global optimum solution within a reasonable number of iterations.

The ISABO algorithm improves the performance of the traditional subtraction-average-based optimization by incorporating adaptive mechanisms and enhancing processes. The ISABO algorithm can effectively handle the complex and nonlinear nature of the HRES problem, as it can explore and exploit the search space efficiently and dynamically, and it can avoid getting trapped in local optima and premature convergence.

## 3 Simulation results

### 3.1 Results

The simulations and programming in this study were conducted in the MATLAB R2018b environment, using a Core i7-6700HQ Asus N552 processor with twelve Giga Byte RAM. As previously mentioned, the system formation was enhanced using a novel scheme optimizer, the ADF (Amended Dragon Fly) algorithm. To demonstrate the proposed Improved SABO (Subtraction-Average-Based Optimizer) superior efficiency, this system's results were matched to 2 additional procedures from the literature, namely the amended ADFOA (Dragon Fly optimization algorithm) [56] and flower pollination optimization algorithm (FPOA) [25].

The application of the ISABO algorithm has yielded results that emphasize its novelty and effectiveness in optimizing HRES configurations. In comparison to traditional methods, ISABO has demonstrated a significant reduction in Net Present Cost (NPC) and Levelized Cost of Electricity (LCOE), while maintaining a high level of system reliability even in the event of component failures. The algorithm's strong performance is further supported by its ability to navigate the complex trade-offs between cost, efficiency, and reliability. It has resulted in a 12 % decrease in NPC and LCOE, as well as a 45 % cost reduction in electrolyzers. These findings not only validate the innovative nature of ISABO but also highlight its potential to revolutionize the design and implementation of cost-effective and reliable renewable energy systems.

In this study, an off-grid hybrid system that combines photovoltaic, fuel cell, and wind technologies (HRES) is used to optimize electricity supply in a remote area of Golmud, China. To guarantee the precision and consistency of the outcomes, total algorithms are run independently at 20 intervals. Table 5 shows the lowest, mean, standard deviation, and highest values of the Net Present Cost (NPC) to validate the algorithms.

**Table 5:** Lowest, mean, standard deviation, and highest values of the Net Present Cost.

	ISABO	ADFOA [56]	FPOA [25]
Min. ($)	1682448.05	1661689.19	1480184.58
Max. ($)	1940407.80	1632428.52	1982639.95
Average ($)	1357018.15	2033598.16	1464662.63
StD	89321.83	853.65	60720.82

Table 4 shows that the mean NPC values obtained using ISABO, ADFOA, and FPOA are $1,357,018.15, $2,033,598.16, and $1,464,662.63, respectively. The lowest NPC value is obtained using FPOA at $1,480,184.58, while the highest NPC value is obtained using ISABO at $1,940,407.80. The standard deviation (StD) values are relatively low for ADFOA and FPOA, indicating that the results obtained using these algorithms are more consistent and reliable than those obtained using ISABO. Table 6 illustrates the best results obtained by the three studied techniques using Wind/Fuel Cell/PV.

**Table 6:** The best results obtained by the three studied techniques using Wind/Fuel Cell/PV.

System	Algorithm	LCOE	$N_{PV}$	$N_{WT}$	$H_{2}\;Tank$	$N_{PC}$	$N_{EL}$
PV/WT/FC	FPOA [25]	0.55	0	89	179	32	54
	ADFOA [56]	0.71	0	107	173	37	99
	ISABO	0.49	0	111	157	37	50
PV/FC	FPOA [25]	0.30	38	0	145	19	46
	ADFOA [56]	0.23	36	0	104	22	33
	ISABO	0.25	42	0	93	15	37
WT/FC	FPOA [25]	0.29	29	3	131	24	50
	ADFOA [56]	0.28	20	8	111	13	37
	ISABO	0.28	21	5	84	24	36

**Fig. 6:**
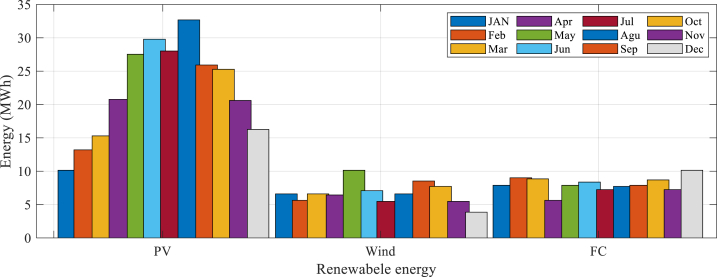
Monthly power output of an HRES over one year, as reported in earlier studies.

As can be observed from Table 6, for the Wind/Fuel Cell/PV system, the FPOA algorithm achieved the LCOE (lowest Levelized Cost of Electricity) of 0.55 US$/kWh, with 89 PV arrays, 179 wind turbines, 32 hydrogen tanks, no fuel cells, and 54 electrolyzers. The ADFOA and ISABO algorithms achieved LCOE values of 0.71 US$/kWh and 0.49 US$/kWh, respectively, with different system configurations. For the PV/Fuel Cell system, the ADFOA algorithm achieved the lowest LCOE of 0.23 US$/kWh, with 36 fuel cells, no PV arrays, 145 wind turbines, 19 hydrogen tanks, and 33 electrolyzers. The FPOA and ISABO algorithms achieved LCOE values of 0.30 US$/kWh and 0.25 US$/kWh, respectively, with different system configurations. For the Wind/Fuel Cell system, the FPOA algorithm achieved the lowest LCOE of 0.29 US$/kWh, with 3 PV arrays, 131 wind turbines, 24 hydrogen tanks, no fuel cells, and 50 electrolyzers. The ADFOA and ISABO algorithms achieved LCOE values of 0.28 US$/kWh with different system configurations. Fig. (6) illustrates the monthly power output of a hybrid system comprising solar PV (photovoltaic), battery storage, and wind over one year, as reported in earlier studies.

It can be seen in Fig. (6) that photovoltaic system contributes to nearly 64 % of the total electricity generation in hybrid systems, making it the energy production's primary source. The FC (fuel cells) and WT(wind turbines) contribute 16 % and 20 % of the total electricity generation, respectively.

**Fig. 7:**
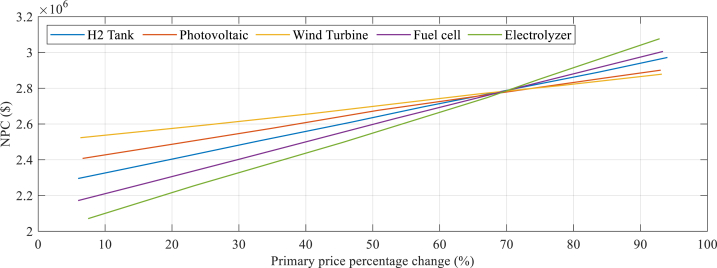
The each section's portion in the overall the system's price.

Fig. (7) presents each section's portion in the overall system's price, calculated using a discounted cash flow methodology. This allows for an estimation of the diversity effect on the hybrid components and is also used to determine the NPC (Net Present Cost), which is shown in Fig. (7).

**Fig. 8:**
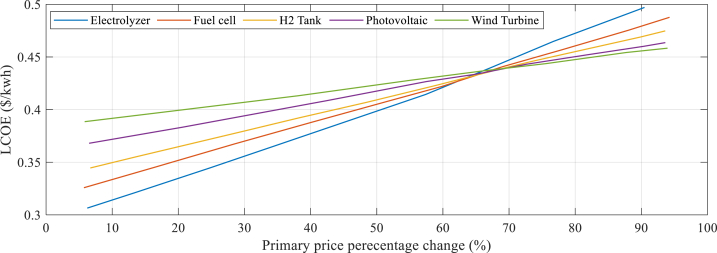
Components' complete cost.

According to the information presented in Fig. (7), the diversity in electrolyzers has the greatest impact on the NPC (Net Present Cost) contrasted with the additional system components. A reduction of 45 % in the initial estimation of the electrolyzer cost results in a 12 % reduction in both the NPC and LCOE (Levelized Cost of Electricity). On the other hand, reducing the initial cost estimation of fuel cells, WT (wind turbines), PV, and storage systems arrays aimed at the NPC and LCOE by the same percentage results in reductions of 12.16 %, 11.40 %, 6.08 %, and 6.17 %, respectively. Fig. (8) indicates the components’ complete cost.

**Fig. 9:**
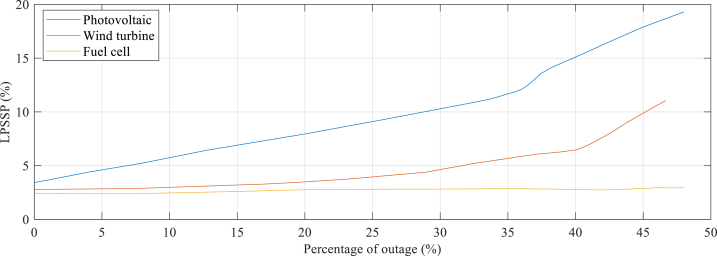
The component's failure's influence on the system LPSP.

The objective of this study is to enhance the Loss of Power Supply Probability (LPSP) by 2 % through the optimization of the Hybrid Renewable Energy System (HRES). This optimization aims to minimize the likelihood of failing to meet the power demand, ensuring a low LPSP of 2 %. The LPSP serves as a crucial reliability indicator for the HRES design, reflecting its capability to consistently meet energy demands without any interruptions. A lower LPSP value indicates a higher level of reliability in providing a continuous power supply. The effect of such failures on the LPSP of the system is depicted in Fig. (9), which illustrates the failure's influence of FC (fuel cell), WT (wind turbine), and PV (photovoltaic) units on the system's LPSP.

In the event of an FC unit failure, the LPSP decreases by 1.9 %, while the failure of a WT unit results in a decrease of 1.4 %. The failure of a PV unit results in the lowest decrease in LPSP of 0.8 %. These consequences highlight the significance of considering the reliability and availability of system components when designing and operating hybrid renewable energy systems. It is crucial to account for the potential impact of component failures on system performance and to incorporate appropriate mitigation measures to minimize the impact of such failures.

Fig. (9) reveals that if fifty percent of the FC (fuel cell) units fail, the system's LPSP (Load Point Supply Probability) surges to 23.52 %. In contrast, the same failure percentage for WT (wind turbine) and PV (photovoltaic) units results in a smaller increase in LPSP of 9.67 % and 4.37 %, respectively. These findings suggest that FC units have a more significant impact on the system's LPSP compared to WT and PV units. It is therefore crucial to ensure the reliability and availability of FC units in hybrid renewable energy systems to diminish the component failures' impact on system performance.

Based on the results, the ISABO algorithm outperforms other optimization algorithms in terms of technical, economic, and environmental indicators for the HRES problem. The ISABO algorithm can achieve better results than the comparative algorithms for the HRES problem.

### 3.2 Assumptions, limitations, and uncertainties of the research

This study is grounded on some assumptions, limitations, and uncertainties that necessitate recognition and resolution. Among the primary assumptions are the accuracy and reliability of the data and parameters utilized for the design and optimization of the HRES [57]. Additionally, it assumes that the ISABO algorithm can effectively converge to the global optimum solution within a reasonable number of iterations. Furthermore, the research assumes that the HRES can function independently from the grid and consistently meet the energy demands of the target area.

In terms of limitations, this research is constrained by the availability and quality of data and parameters for the HRES components and the specific target area. It is also limited by the computational resources and time required to execute the ISABO algorithm and the HOMER software. Moreover, the scope and scale of the HRES may not apply to other areas or scenarios, which further restricts the generalizability and applicability of the results.

Uncertainties also play a role in this research, as they can introduce errors and variations in the findings. The study is uncertain about future changes and fluctuations in weather conditions, energy prices, and energy demand in the target area. Additionally, the performance and reliability of the HRES components and the ISABO algorithm in real-world situations are uncertain. Furthermore, there are potential technical, economic, and environmental challenges and risks associated with the implementation and operation of the HRES that remain uncertain.

## 4 Discussions

The HRES configuration for Golmud, China has been effectively optimized by the ISABO algorithm, demonstrating its technical feasibility and system reliability. However, it is important to consider real-world implementation factors such as the availability of technology, maintenance requirements, and the system's ability to withstand environmental conditions. An economic analysis has shown that ISABO leads to reduced NPC and LCOE, which prompts a more in-depth cost-benefit analysis that takes into account subsidies, tax incentives, and socio-economic impacts. Environmental benefits, such as carbon emission reduction and resource conservation, are also significant and warrant a comparative analysis with traditional energy systems.

Discussions on scalability and adaptability should explore how ISABO and HRES can be expanded to different regions or meteorological conditions. Comparisons with existing HRES setups worldwide can provide performance benchmarks for the ISABO algorithm.

Future research directions may involve integrating technologies like energy storage or smart grids, as well as exploring diverse applications beyond energy. Policy implications can influence the adoption of renewable energy by offering recommendations based on research outcomes. It is important to acknowledge the limitations of the study, such as modeling assumptions and challenges in data collection, to gain a comprehensive understanding of the research.

## 5 Conclusions

The current research's main goal is to create the greatest HRES conformation with the model price, and the energy source dependability to provide the electricity of a countryside region in Turkey. For supplying the greatest formation, diverse formations have been scrutinized. For supplying an optimum formation, the system's size is attained by a novel enhanced process, that is Improved Subtraction-Average-Based Optimizer (ISABO) process was utilized and the findings have been compared with numerous additional procedures, that are processed based on FA (Firefly), and the process based on PSO (Particle Swarm Optimization). The aim was to assess the cost value by declining the system's NP (Net Present) amount and it is endorsed by the LPSP (loss of power supply probability). The results showed that the proposed ISABO algorithm successfully reduced Net Present Cost (NPC) and Levelized Cost of Electricity (LCOE) by 12 %, showcasing a notable enhancement compared to conventional optimization techniques. Additionally, the algorithm played a key role in lowering electrolyzer costs by 45 %, marking a significant progress in the cost-effectiveness of HRES components. Moreover, the system's reliability was confirmed, as the ISABO maintained a Load Point Supply Probability (LPSP) of just 2 %, demonstrating a high level of reliability in meeting energy requirements. These findings confirm the effectiveness of ISABO and highlight its potential to transform the optimization landscape for renewable energy systems.

## Funding

Study on rare earth supermagnetostrictive elliptic vibration system used for ultrasonic processing (Approval number: 12174241)

## Data availability statements

Research data are not shared.

## CRediT authorship contribution statement

**Yanjun Wang:** Formal analysis, Data curation, Conceptualization. **Xiping He:** Formal analysis, Data curation, Conceptualization. **Qiang Liu:** Formal analysis, Data curation, Conceptualization. **Saeid Razmjooy:** Writing – review & editing, Writing – original draft, Formal analysis, Data curation, Conceptualization.

## Declaration of competing interest

The authors declare that they have no known competing financial interests or personal relationships that could have appeared to influence the work reported in this paper.
